# Human Prosocial Preferences Are Related to Slow-Wave Activity in Sleep

**DOI:** 10.1523/JNEUROSCI.0885-23.2024

**Published:** 2024-03-11

**Authors:** Mirjam Studler, Lorena R. R. Gianotti, Janek Lobmaier, Angelina Maric, Daria Knoch

**Affiliations:** ^1^Department of Social Neuroscience and Social Psychology, Institute of Psychology, University of Bern, Bern 3012, Switzerland; ^2^Department of Neurology, University Hospital Zurich, University of Zurich, Zurich 8091, Switzerland

**Keywords:** cooperation, neural trait, prosociality, public goods game, sleep slow-wave activity, temporoparietal junction

## Abstract

Prosocial behavior is crucial for the smooth functioning of the society. Yet, individuals differ vastly in the propensity to behave prosocially. Here, we try to explain these individual differences under normal sleep conditions without any experimental modulation of sleep. Using a portable high-density EEG, we measured the sleep data in 54 healthy adults (28 females) during a normal night's sleep at the participants' homes. To capture prosocial preferences, participants played an incentivized public goods game in which they faced real monetary consequences. The whole-brain analyses showed that a higher relative slow-wave activity (SWA, an indicator of sleep depth) in a cluster of electrodes over the right temporoparietal junction (TPJ) was associated with increased prosocial preferences. Source localization and current source density analyses further support these findings. Recent sleep deprivation studies imply that sleeping enough makes us more prosocial; the present findings suggest that it is not only sleep duration, but particularly sufficient sleep depth in the TPJ that is positively related to prosociality. Because the TPJ plays a central role in social cognitive functions, we speculate that sleep depth in the TPJ, as reflected by relative SWA, might serve as a dispositional indicator of social cognition ability, which is reflected in prosocial preferences. These findings contribute to the emerging framework explaining the link between sleep and prosocial behavior by shedding light on the underlying mechanisms.

## Significance Statement

Sleep deprivation reportedly hampers prosocial behavior. Yet, sleep loss is not a regular occurrence. We studied participants without experimentally manipulating their sleep and conducted polysomnography along with a prosocial economic task. We found that higher relative slow-wave activity (an indicator of sleep depth) in the right TPJ—a brain region involved in social cognition—is associated with increased prosociality. This demonstrates a novel link between deep sleep neural markers and prosocial preferences. Furthermore, our study provides evidence about a possible neural mechanism that underlies the behavioral findings of previous studies on sleep deprivation and prosocial behavior. Our findings highlight the significance of sleep quality in shaping prosociality and the potential benefits of interventions targeting sleep quality to promote social capital.

## Introduction

Prosocial behavior is of vital importance for holding our society together. Yet, the propensity to exhibit prosocial behavior is characterized by vast individual differences (Fischbacher et al., 2001; Declerck and Boone, 2018; Thielmann et al., 2020). However, within a person, prosocial behavior has been shown to be stable over time and across different situations (Carlsson et al., 2014; Peysakhovich et al., 2014). Here, we aim to explain individual differences in prosocial preferences using a stable neural trait, namely, the topographical distribution of slow-wave activity (SWA) during sleep.

Recent evidence shows a striking relationship between the amount of sleep we get and social functioning (Holbein et al., 2019; Ben Simon et al., 2020, 2022; Clark and Dickinson, 2020). Specifically, sleep deprivation has been associated with reduced altruism, trustworthiness, trust, and helping behavior (Anderson and Dickinson, 2010; Dickinson and McElroy, 2017; Ben Simon et al., 2022).

There have been two prevailing attempts to explain why prosocial behavior is negatively impacted by sleep deprivation (for a review, see Dorrian et al., 2019). One possible reason may be that sleep deprivation hampers social cognition abilities (Guadagni et al., 2014, 2017; Ben Simon and Walker, 2018; Ben Simon et al., 2020, 2022). Support for this explanation is provided by Ben Simon et al. (2022) who found reduced activity in key nodes of the social cognition network [temporoparietal junction (TPJ), mPFC, precuneus] and a decrease in the desire to help others under conditions of sleep loss.

A second attempt to explain reduced prosocial behavior after sleep deprivation stems from the idea that sleep deprivation interferes with self-control abilities, deliberative thinking, and executive functioning (Anderson and Dickinson, 2010; Dickinson and McElroy, 2017; Holbein et al., 2019). These functions are crucial for forming prosocial behavior (Wyss and Knoch, 2022) and are associated with prefrontal brain regions (Knoch and Fehr, 2007; Hare et al., 2009), which are particularly affected by sleep deprivation (Harrison et al., 2000; Thomas et al., 2003; Killgore et al., 2008; Groeger et al., 2014).

The studies reported above nicely demonstrate that artificially limiting sleep affects prosocial behavior. However, no study has yet examined how **e**lectrophysiological measures of sleep under normal conditions (i.e., without experimental manipulation) are linked to prosocial behavior. In the present study, we hence examine how the processes happening in the sleeping brain relate to the vast individual differences in prosocial preferences. To do so, we look at the trait-like characteristics of the sleeping brain in individuals who habitually sleep between 7 and 8 h every night. Specifically, we measured SWA during sleep. SWA is a major EEG hallmark of deep sleep and an objective measure of sleep depth. We then correlated the topographical distribution of SWA with individual prosocial preferences**.**

The topographical distribution of SWA shows local differences that are highly stable within but vary between individuals (Finelli et al., 2001; Lustenberger et al., 2017) and is therefore unique to each person (Markovic et al., 2018; Rusterholz and Achermann, 2011). Here, we investigated the association of the relative SWA topography with individual differences in prosocial preferences.

The present study is designed to scrutinize whether the topographical distribution of relative SWA under normal sleep conditions explains individual differences in prosocial preferences. To capture individual differences in prosocial preferences, we employed a public goods game (PGG). To comprehensively measure prosocial preferences, it is necessary to also measure what people believe others would contribute (see Materials and Methods, Measurement of prosocial preferences for a detailed explanation). As this is the first study of its kind, we do not have any a priori hypotheses. However, based on the sleep deprivation studies mentioned above, we may tentatively expect SWA differences in the areas involved in impulse control and deliberate thinking, such as the PFC (Dickinson and McElroy, 2017; Holbein et al., 2019) and/or the social cognition network including the TPJ, mPFC, and precuneus (Ben Simon et al., 2022).

## Materials and Methods

### Participants

We calculated the sample size required to achieve 80% power to detect significant correlations (α = 0.005) using G*Power 3.1.9.7 (*F* tests, linear multiple regression; Faul et al., 2007). Based on our previous sleep study on neural traits and risk preferences (Studler et al., 2022) and based on previous studies on neural traits and economic preferences during wakefulness (Gianotti et al., 2009; Knoch et al., 2010; Baumgartner et al., 2013), we assumed a medium effect size of *f*^2 ^= 0.25. The power analysis yielded a recommended sample size of 58 participants. Since we performed the sleep EEG recordings at the participants' homes without the constant supervision of an experimenter, we expected dropouts because of technical issues. We therefore recruited a total of 62 healthy right-handed participants.

Eight participants were excluded due to noncompliance to the study protocol (*n* = 2) or the missing EEG data (*n* = 6), leaving 54 participants (mean age, 21.5 years old; SD age, 2.0 years; 28 females) for analyses. All participants were informed of their right to discontinue their participation at any time and gave written informed consent. Participants received 155 Swiss francs (CHF 155; CHF 1 ≈ $ 1 US) as compensation for participating in the morning after the night of sleep, in addition to the money earned in the behavioral task, which depended on their own and others' behavior (see Measurement of prosocial preferences). The earnings from the behavioral assessment were paid immediately after completing the PGG. Ethical approval for this experiment was provided by the local ethics committee and adheres to the principles of the Declaration of Helsinki.

### Procedure

All recruited participants were screened before the experiment to meet the following inclusion criteria: right-handedness (Chapman and Chapman, 1987); self-reported good sleepers with a habitual sleep duration of 7–8 h per night (Pittsburgh Sleep Quality Index, <5; Buysse et al., 1989); normal sleepiness index (Epworth Sleepiness Scale, <10; Johns, 1991); no extreme chronotype (Munich Chronotype Questionnaire, >2 and <7; Roenneberg et al., 2003); no current or past history of neurological, psychiatric, or sleep disorders; no drug nor alcohol abuse; no regular medication intake; normal weight; and no traveling across more than two time zones within the last 30 d before the experiment. Additionally, participants were asked about their regular caffeine, alcohol, and nicotine consumption. Because women's sleep quality can be influenced by their menstrual cycle phase (Baker and Driver, 2004), we controlled for the cycle phase using the forward counting method. Naturally cycling women were not invited during their estimated fertile days or during the first 2 d of their menstrual cycle. Women using hormonal contraception were not invited during pill-free intervals.

**Figure 1.**
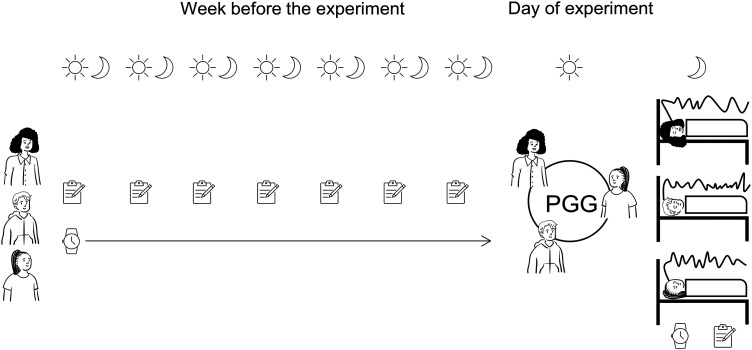
Study design. One week before the experiment, participants were instructed to maintain a regular sleep–wake rhythm. They were given an actigraph to objectively monitor their sleep–wake rhythm. During this week, participants completed sleep and consumption diaries to ensure adherence to the study protocol. On the experiment day, participants came to the laboratory in groups of three to play the PGG. Afterward, they were fitted with a high-density portable EEG system and sent home where the sleep EEG was recorded during the following night.

One week before the experiment, participants were invited to the laboratory where they received detailed instructions. We asked participants to keep a regular sleep–wake rhythm adjusted to their habitual bedtimes (sleep duration of 7–8 h) and to refrain from daytime napping throughout the week before the experiment (Fig. 1). Participants were also asked to limit their caffeine consumption to two units/day (one unit, caffeine content of one cup of coffee) and their alcohol consumption to one standard drink/day (one standard drink, one beer; 350 ml = 10 g ethanol). Smokers were told to adhere to their habitual nicotine consumption. Each participant received a triaxial accelerometer (GENEActiv, Activinsights) to wear on their nondominant hand. Actigraphy is a validated objective measure of sleep behavior (de Souza et al., 2003; Marino et al., 2013), discerning sleep from being awake based on motion. The single-use straps ensured that participants did not remove the actigraph during the week of actigraphy measurement. Additionally, we also used sleep diary and consumption diary entries to confirm their adherence to the study protocol. Finally, participants were given a chest harness with a sham amplifier to simulate the wearing of the portable high-density EEG system. We asked participants to sleep with the chest harness and the sham amplifier to find the optimal amplifier position for the recording night.

On the day of the experiment, participants were asked to refrain from extensive exercise or visiting the sauna to avoid post sweating. Participants came to the laboratory in groups of three to play the PGG (Fig. 1). To ensure anonymity, participants were invited to three different floors of the building and were accompanied one after the other to the cubicles they were randomly assigned to. After this, participants were fitted with the portable high-density EEG system and were sent home, where they continued with their habitual routine. Shortly before bedtime, the experimenters visited the participants at home to check and, if needed, correct the impedances of the electrodes and start the recording (Fig. 1). Participants also underwent an implicit association task, but this task was irrelevant to the present study.

### Measurement of prosocial preferences

In each experimental session, the three participants sat in their cubicles with interconnected computer terminals where they could make their decisions in complete anonymity from the other two participants. For the measurement of prosocial preferences, we used the PGG. Each participant was endowed with 20 points (1 point = CHF 0.5) and faced the decision (one-shot) to either keep their endowment or contribute all or part of it to a public good (0–20 points). Each point contributed was doubled by the experimenter, and the resulting sum was divided equally among the three participants. Hence, each point contributed increased the aggregate group payoff while diminishing the contributing individual's payoff. Immediately after the contribution decision, participants reported their belief about the average contribution of the other two participants (0–20 points). This was done because several studies have shown that the amount people contribute to the public good is influenced by what they believe the other participants will contribute (Neugebauer et al., 2009; Fischbacher and Gächter, 2010). For example, one participant might contribute half of their endowment because they assume that the other participants would contribute a comparable amount. In contrast, a different participant might also contribute half of the endowment because they simply find this the fairest decision. In the latter case, the participant contributes according to their prosocial inclination without strategically considering the decisions of the other players, while in the first case, the contribution is conditional on what other players are expected to contribute. So, even though in both cases the two exemplified participants contributed the same amount, this does not reflect the same level of prosocial preferences. To accommodate the differences in prosocial preferences, we hence asked our participants after their contribution decisions what they believed the other players had contributed. To get a measure that comprehensively measures prosocial preferences, we generated a difference score by subtracting the value of the participants' beliefs from their own contributions (contribution-minus-belief score).

The participants' final payoff in the PGG consisted of the earnings they gained from the public good and the points they had kept for themselves. Participants received detailed written instructions before the task, including information about the calculation of the final payoff. Comprehension trials ensured their understanding by asking the participants to calculate payoff distributions in different scenarios.

### Sleep EEG recording

High-density portable EEG (LiveAmp 64, Brain Products) with 64 electrodes (actiCAP, EASYCAP), including three electrooculogram and two submental electromyogram channels, were continuously recorded during the nighttime sleep episode. Two additional electrodes were used as recording reference (Cz) and as ground (AFz). The electrical signals were recorded with a sampling rate of 500 Hz (third-order low-pass filter at 131 Hz). Impedances were kept below 25 kΩ. For each participant, lights-off and wake-up times were determined according to his or her habitual sleep time.

### Sleep EEG preprocessing

The data were offline bandpass filtered between 0.5 and 40 Hz. Sleep was visually scored according to the standard criteria (Berry et al., 2018). The data from the seven channels required for sleep scoring only (two electromyogram, three electrooculogram, and two mastoid channels) were then excluded, leaving a total of 59 electrodes for further analyses. The following sleep parameters were extracted from the sleep stage scoring: total sleep time (i.e., the objective sleep quantity), sleep efficiency (proportion of total time in bed spent asleep), wake after sleep onset (length of periods of wakefulness occurring after sleep onset), and percentage of the total sleep time spent in each sleep stage [N1, N2, N3, and rapid eye movement (REM)].

Bad channels were individually identified by the visual inspection of time–frequency plots and spectrograms of the whole night. On average, 5.75% of channels were deemed bad and were excluded, if problematic at any time of the night. The remaining signals were then rereferenced to the average of all good channels. The power density spectra were then calculated for 30 s epochs using the fast Fourier transformation (5 s subepochs, Hanning window, no overlap). Artifacts were excluded semiautomatically, whenever the power exceeded a threshold based on a moving average over epochs for the frequency bands 0.8–4.6 and 20–40 Hz (Buckelmüller et al., 2006).

### SWA distribution maps and source localization

SWA in the range between 0.8 and 4.6 Hz in sleep stages N2 and N3 was computed for further analyses. SWA values from the excluded channels were interpolated using spherical linear interpolation (Delorme and Makeig, 2004). Individual SWA distribution maps were normalized to the mean values across all electrodes, yielding relative SWA distribution maps (Finelli et al., 2001). Relative SWA was log-transformed before statistical analyses in order to approach a normal distribution.

A source localization analysis was performed using the standardized low-resolution brain electromagnetic tomography (sLORETA) method (Pascual-Marqui, 2002). The sLORETA algorithm has been used in many sleep EEG studies (Bersagliere et al., 2017; Moffet et al., 2020; Castelnovo et al., 2022) and has been applied to estimate the cortical localization of NREM sleep sources (Siclari et al., 2018; Fernandez Guerrero and Achermann, 2019; Stephan et al., 2021). Using the manual regularization method in the sLORETA software, we selected the transformation matrix with the signal-to-noise ratio set to 10. sLORETA images were then log-transformed before the statistical analyses. Additionally, we calculated the current source density (CSD) maps. The CSD maps were computed from artifact-free EEG data using the Laplacian transformation. The CSD maps are effectively reference-free (Kayser and Tenke, 2015). CSD power in the range between 0.8 and 4.6 Hz (CSD SWA) was then calculated in sleep stages N2 and N3 using the fast Fourier transformation. Individual CSD SWA distribution maps were normalized to the mean values across all electrodes, yielding relative CSD SWA distribution maps. Relative CSD SWA was log-transformed before statistical analyses in order to approach the normal distribution. An electrode-wise Pearson’s correlation approach was taken to identify scalp regions whose relative CSD SWA during an entire night of sleep under normal conditions correlated with the contribution-minus-belief score.

### Statistical analyses

In the main analyses, as a first step, an electrode-wise Pearson’s correlation approach was taken to identify scalp regions whose relative SWA during an entire night of sleep under normal conditions correlated with the contribution-minus-belief score. To correct for multiple comparisons, we applied a statistical nonparametric mapping using a suprathreshold cluster analysis (Nichols and Holmes, 2001; Huber et al., 2004). For each permutation, the maximal cluster size of the neighboring electrodes reaching an *r* value above the critical value was counted and used to build a cluster-size distribution. The 95th percentile was defined as the critical cluster-size threshold. To better describe and visualize the result of this analysis, for each participant, relative SWA was then averaged in the significant cluster. As a second step, we estimated the intracerebral sources that gave rise to the significant cluster. For our voxel-by-voxel Pearson’s correlation analyses, we created a 15 mm sphere centered on MNI coordinates of the right TPJ (right TPJ, *x* = 54, *y* = −52, *z* = 32; Krall et al., 2015). We corrected for multiple testing of all of the 59 voxels via a nonparametric randomization approach (Nichols and Holmes, 2001).

As additional analyses, we repeated the electrode-wise Pearson’s correlation approach between SWA and the contribution-minus-belief score for the individual sleep cycles. Sleep cycles were defined according to an adaptation of Feinberg and Floyd's criteria (Feinberg and Floyd, 1979; Jenni and Carskadon, 2004; Kurth et al., 2010). For the calculation of relative SWA in individual sleep cycles, we normalized SWA values to the mean values across all electrodes within each cycle.

## Results

### Behavioral results and sleep parameters

**Figure 2.**
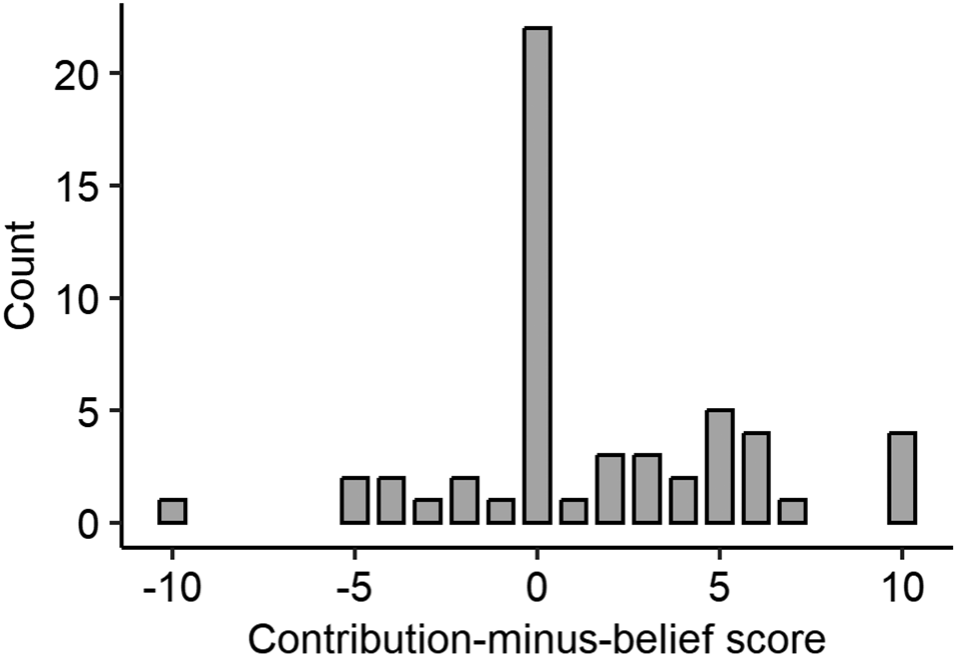
Histogram depicting the distribution of the contribution-minus-belief score among all participants.

As illustrated in Figure 2, we observed large interindividual differences in prosocial preferences. The contribution-minus-belief score varied from −10 to 10 (*M* = 1.56, SD = 4.03). Sleep parameters were within the expected range for this age group (Table 1).

**Table 1.** Mean with 95% CIs for total sleep time, sleep efficiency, wake after sleep onset, and duration of sleep stages for the total sample (*N* = 54)

	Total sleep time (min)	Sleep efficiency (%)	Wake after sleep onset (min)	Duration of sleep stages (% of total sleep time)			
				N1	N2	N3	REM
Mean	431.6	93.1	21.5	7.7	46.3	24.7	21.3
95% CIs	422.6–441.0	92.2–93.9	18.2–24.9	6.7–8.7	44.8–47.9	23.2–26.3	20.2–22.3

### Brain results

**Figure 3.**
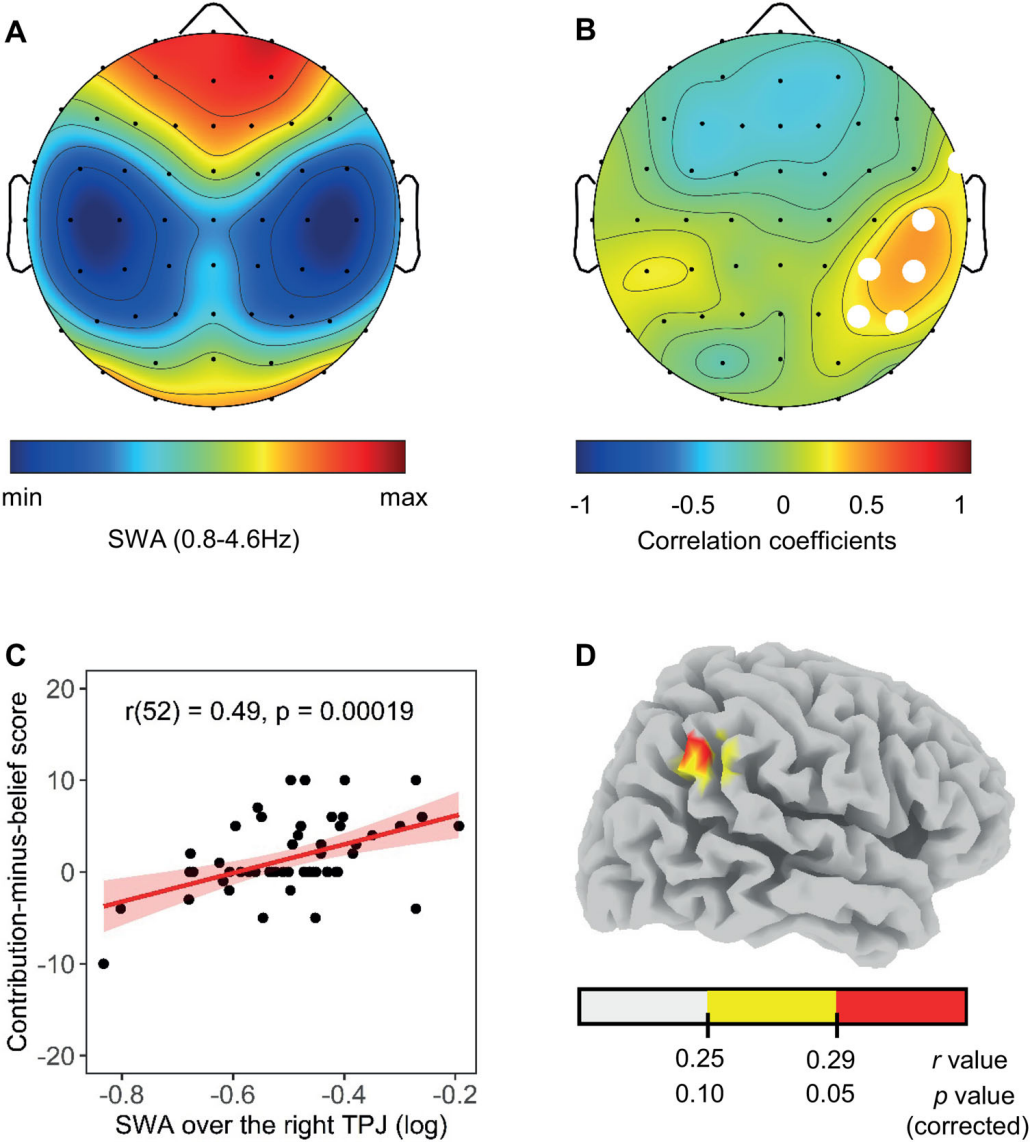
Topographical distribution of relative SWA (0.8–4.6 Hz) and its correlation with prosocial preferences. ***A***, Topographical distribution of relative SWA (average over all participants). SWA values at every electrode were normalized in relation to the average SWA over all electrodes of a participant. The dark blue to dark red colors indicate minimal (45%) to maximal (173%) SWA. ***B***, Statistical scalp distribution of *r*-coefficients between relative SWA and prosocial preferences. The blue areas indicate negative correlations; the red areas indicate positive correlations. The white dots indicate electrodes with significant correlations (*p* < 0.05, corrected for multiple testing with a suprathreshold cluster analysis). The black dots indicate the position of the 59 electrodes. ***C***, Scatterplot of the positive correlation between mean relative SWA in the significant cluster over the right TPJ and prosocial preferences (including regression line and confidence interval 95%). ***D***, Relationship between SWA current density in the right TPJ and prosocial preferences. Locations of the voxels that showed significant correlations are indicated in red (*p* < 0.05, corrected) and yellow (*p* < 0.10, corrected).

In the main analysis, we checked whether individual differences in the topographical distribution of relative SWA in N2 and N3 (Fig. 3*A*) during an entire night of sleep explain individual differences in prosocial preferences. We found robust and significant positive associations in a cluster of six electrodes placed over the right TPJ (C6, CP4, CP6, FT8, P4, P6, *p* < 0.05, corrected for multiple testing; Fig. 3*B*). The correlation between mean relative SWA in the significant cluster and prosocial preferences resulted in a correlation coefficient of 0.49 (df = 52), *p* = 0.00019, *R*^2 ^= 0.24 (Fig. 3*C*). Crucially, partialling out participants' total sleep time or time spent in deep sleep (i.e., sleep stages N2 and N3) did not affect the relation between relative SWA over the right TPJ and prosocial preferences (*r*_(51)_ = 0.49, *p* = 0.00019, *R*^2 ^= 0.24; *r*_(51)_ = 0.50, *p* = 0.00016, *R*^2 ^= 0.25). Thus, the positive correlation between relative SWA over the right TPJ and prosocial preferences was independent of the quantity of sleep. Moreover, partialling out the participants' age and gender also did not affect the relationship between relative SWA over the right TPJ and prosocial preferences (*r*_(50)_ = 0.49, *p* < 0.00001, *R*^2 ^= 0.24).

**Figure 4.**
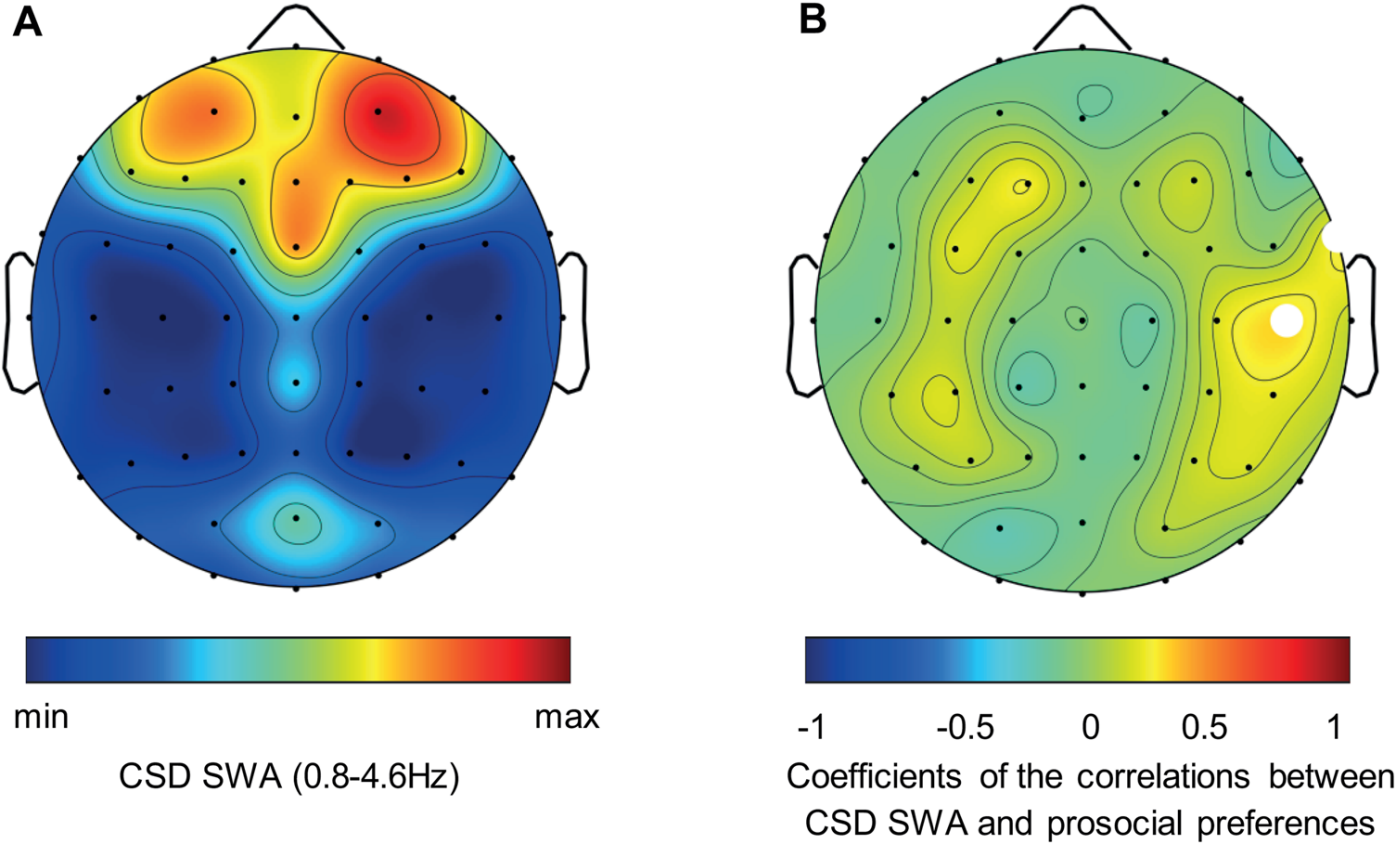
Topographical distribution of relative CSD SWA (0.8–4.6 Hz) and its correlation with prosocial preferences. ***A***, Topographical distribution of relative CSD SWA (averaged over all participants). The dark blue to dark red colors indicate minimal (58%) to maximal (195%) CSD SWA. ***B***, Statistical scalp distribution of *r*-coefficients between log-transformed relative CSD SWA and prosocial preferences. The blue areas indicate negative correlations; the red areas indicate positive correlations. The white dots indicate electrodes with significant correlations (*p* < 0.05). The black dots indicate the position of the 59 electrodes.

Since scalp-based correlation maps provide only a rough estimate of regional characteristics, we used sLORETA to estimate the regional specificity of the previous findings. We found three voxels in the right TPJ showing significant positive correlations between SWA current density and prosocial preferences (*p* < 0.05, small volume corrected for multiple testing; MNI coordinates of peak voxel, *x* = 55, *y* = −55, *z* = 45; inferior parietal lobule, BA 40; Fig. 3*D*, and for the CSD results, see Fig. 4).

**Figure 5.**
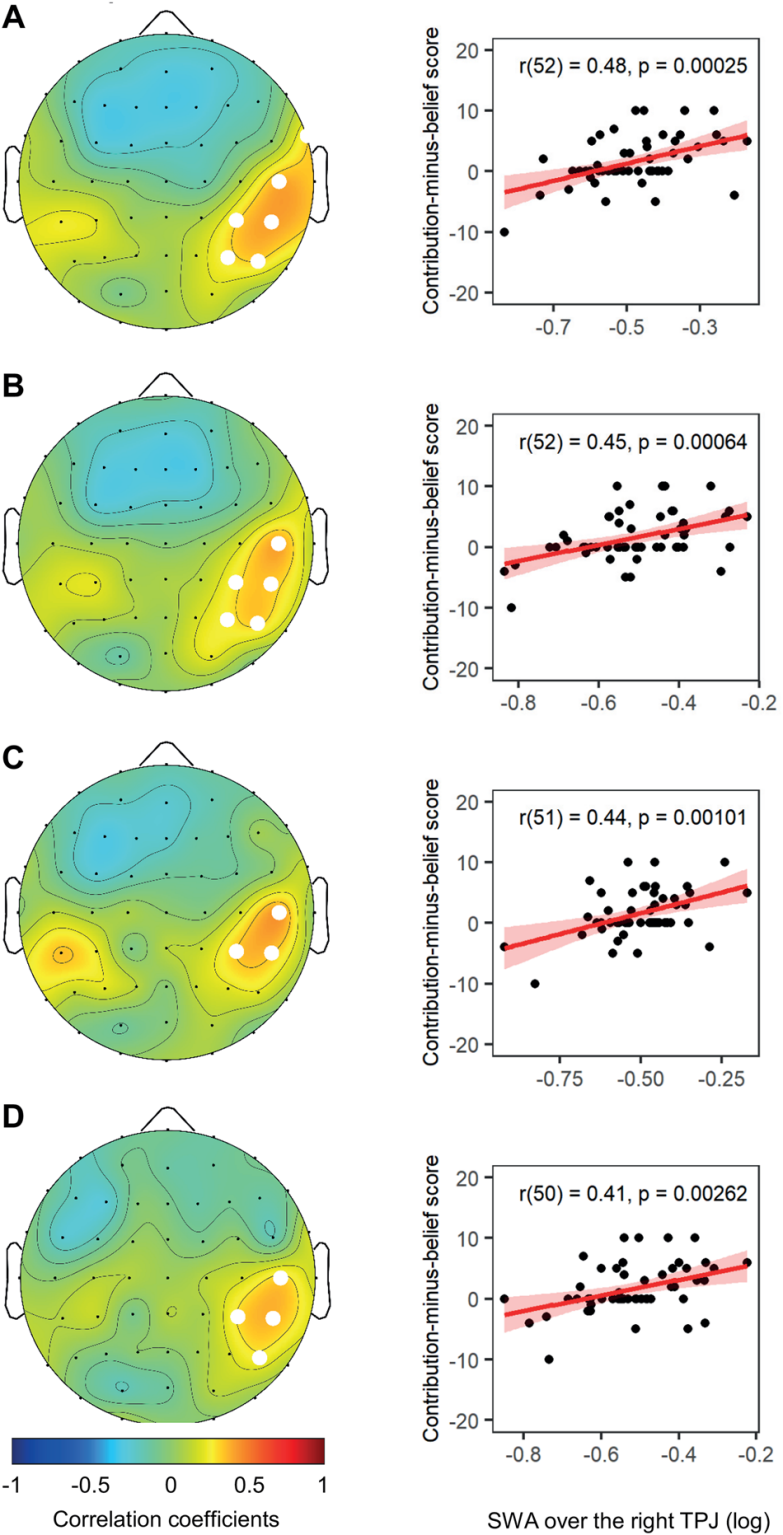
Relationship between relative SWA and prosocial preferences for Sleep Cycle 1 (***A***), Sleep Cycle 2 (***B***), Sleep Cycle 3 (***C***), and Sleep Cycle 4 (***D***). The left panels show statistical topographical distributions of correlation coefficients between relative SWA and prosocial preferences. The blue areas indicate negative correlations; the red areas indicate positive correlations. The white dots indicate electrodes with significant correlations (*p* < 0.05) in the cluster of six electrodes identified in the main analysis (Fig. 3*B*). The right panels show scatterplots of the positive correlations between mean relative SWA in the significant cluster over the right TPJ and prosocial preferences (including regression line and confidence intervals 95%).

SWA levels typically decline across a night of sleep (Achermann et al., 1993). As the rate of the decline varies at different cortical areas, averaging SWA over an entire night of sleep might lead to a loss of information. Therefore, we performed additional analyses where we correlated relative SWA over the right TPJ cluster with prosocial preferences separately for all sleep cycles. Not all participants had a fifth sleep cycle; hence, we present analyses from four cycles. The results, presented in Figure 5, demonstrated a highly similar pattern for each of the four cycles compared with the whole night (Fig. 3*B*).

To ensure that the main result was not driven by SWA in the first sleep cycle, when SWA levels are typically the highest, we excluded this cycle in a further analysis and correlated relative SWA of the second, third, and fourth sleep cycles pooled together with prosocial preferences. The result once again shows a significant positive correlation between relative SWA over the TPJ and prosocial preferences (*r*_(52)_ = 0.48, *p* = 0.00021, *R*^2 ^= 0.23).

### Additional analysis

**Figure 6.**
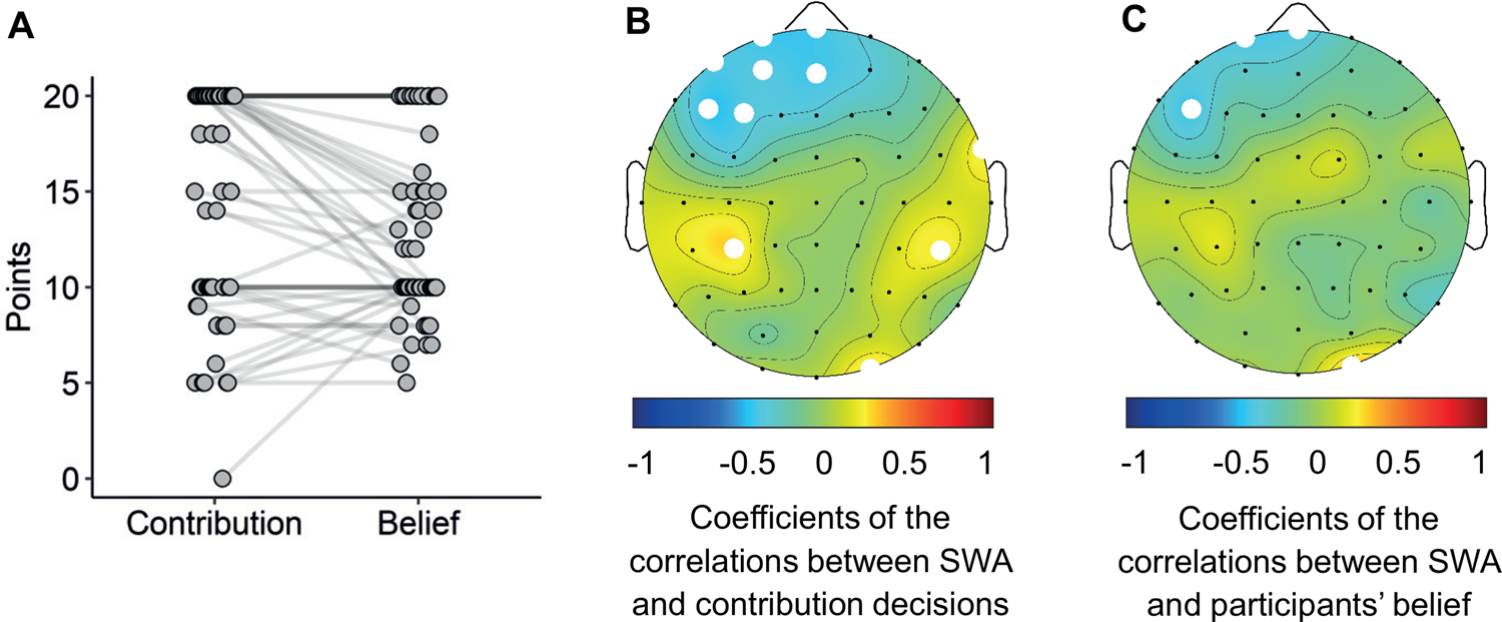
Contribution decisions, beliefs, and correlations with relative SWA. ***A***, Contribution decisions and participants' beliefs about the average contribution of the other participants. Contribution decision (left) and the belief about others’ contribution (right) are depicted for each participant. Contribution decisions and corresponding beliefs per participants are joined by a line. ***B***, Topographical distribution of *r*-coefficients between relative SWA and contribution decisions and (***C***) participants' beliefs. The blue areas indicate negative correlations; the red areas indicate positive correlations. The white dots indicate electrodes with uncorrected significant correlations (*p* < 0.05). The black dots indicate the position of the 59 electrodes.

Our study aimed to investigate how human prosocial preferences are related to SWA during sleep. As mentioned in Materials and Methods, Measurement of prosocial preferences, ample evidence demonstrates that individuals adjust their contributions based on their beliefs about other's contributions (Neugebauer et al., 2009; Fischbacher and Gächter, 2010). Therefore, in the main analyses, we focused on the contribution-minus-belief score, because this measure more accurately reflects prosocial preferences rather than the contribution or belief alone (see Materials and Methods, Measurement of prosocial preferences for a detailed explanation). However, for the sake of completeness, we present additional results separately for the contribution and the belief scores (Fig. 6).

## Discussion

Recent research emphasizes the importance of sleep for prosocial behavior (Holbein et al., 2019; Ben Simon et al., 2020, 2022; Clark and Dickinson, 2020). While this research demonstrates that adverse sleep conditions have negative consequences on people's social behaviors, these studies offer no conclusions on the underlying mechanisms of how sleep impacts prosocial behavior and how individual differences in prosocial inclinations come about. We attempted to better understand the connection between sleep and prosociality by directly looking at the sleeping brain. Rather than experimentally preventing people from sleeping and then looking at their prosocial behavior, we used a portable high-density EEG system to record SWA in self-reported good sleepers during a normal night's sleep. Our results demonstrate an intriguing association between a trait-like sleep characteristic; relative SWA, in the TPJ; and prosocial preferences.

Different attempts have been made to explain why prosocial behavior is negatively impacted by sleep deprivation. One suggested possibility for why sleep deprivation may lead to reduced prosocial behavior is that sleep deprivation hampers self-control, deliberative thinking, and executive functioning (Anderson and Dickinson, 2010; Dickinson and McElroy, 2017; Holbein et al., 2019). A second explanation assumes the involvement of the social cognition network (Ben Simon and Walker, 2018; Ben Simon et al., 2020, 2022). For example, it has been found that the desire to socially interact with others decreases upon sleep loss, while the desire to be alone increases (Ben Simon and Walker, 2018; Axelsson et al., 2020). Other studies have found that sleep loss negatively impacts empathy. For example, Guadagni et al. (2014) demonstrated that one night of total sleep deprivation leads to reduced emotional empathy. Of special interest in the context of the present study is the finding of Ben Simon et al. (2022), who used functional MRI analyses to examine the underlying neural changes in order to explain the association between inadequate sleep and reduced prosociality. They found that, relative to the rested condition, sleep loss was associated with a significant reduction in task-evoked activity within the social cognition network, namely, in the TPJ, the mPFC, mid- and superior temporal sulcus, and the precuneus. So, these authors could nicely demonstrate that the social cognition network functions differently after adverse sleep conditions. In the present study, we go a step further by looking at the activity in the sleeping brain during habitual sleep. We found that more relative SWA in the TPJ is associated with increased prosocial preferences. Our finding hence offers further support for the idea that sleep influences the social cognition network (cf. Ben Simon and Walker, 2018; Ben Simon et al., 2020, 2022).

A large body of evidence looking at the waking brain has consistently linked the task-dependent activation of the TPJ with aspects of social cognition such as mentalizing, perspective-taking or “theory-of-mind” (ToM), self-other distinction, and empathy (Saxe, 2006; Decety and Lamm, 2007; Carter and Huettel, 2013). These aspects of social cognition include understanding and monitoring the mental states of others such as their intentions, beliefs, desires, emotions, and actions and are crucial for prosocial behavior (Frith and Frith, 2007). Various studies have linked the activation in the TPJ with generous choices (Hutcherson et al., 2015; Strombach et al., 2015; Park et al., 2017) and donation behavior (Hare et al., 2010; Van Hoorn et al., 2016). Support for the causal involvement of the TPJ in prosocial behavior and perspective-taking stems from neuro-modulation studies (Li et al., 2020; Hao et al., 2021; Langenbach et al., 2022). Li et al. (2020), for example, demonstrated that increasing the cortical excitability using anodal tDCS over the TPJ increased the participants' charitable giving.

Previous research using a neural trait approach during wakefulness also reported a link between individual differences in prosociality and the TPJ (Morishima et al., 2012; Gianotti et al., 2018, 2019; Baumgartner et al., 2019). For example, a resting-state EEG study found that the task-independent baseline activation in the TPJ is related to interindividual variation in prosocial behavior (Gianotti et al., 2019). Similarly, gray matter volume in the TPJ was positively associated with altruistic choices in a structural MRI study (Morishima et al., 2012). Interestingly, recent studies showed that increased slow-wave density is linked to higher cortical thickness (Dubé et al., 2015).

In the present study, we found a positive correlation between relative SWA in the TPJ and prosocial preferences. SWA is an ideal candidate for capturing individual differences in prosocial preferences. We have several reasons to believe that the SWA measured in our study indeed reflects trait-like differences. During the 7 d before the experiment, sleep and consumption diaries as well as actigraphy were used to confirm the adherence to the study protocol (i.e., regular sleep–wake rhythm, sleep duration of 7–8 h, no daytime napping). This procedure was introduced to minimize possible state effects. In addition, we divided SWA power at every single electrode by the average SWA over all electrodes, resulting in individual topographical distributions indicating relative SWA. Absolute SWA levels (i.e., without normalization) are subject to day-to-day variations and to a decline across the sleep period (state-dependent) and therefore reflect the prevailing sleep–wake history. On the contrary, topographical maps of relative SWA have been shown to be very stable and thus trait-like (Finelli et al., 2001; Rusterholz and Achermann, 2011). Consequently, the fingerprint-like SWA topography has been suggested to reflect neural differences across individuals (Finelli et al., 2001). Also, we ran separate analyses for the individual sleep cycles and in every sleep cycle relative SWA over the right TPJ correlated significantly with prosocial preferences. So, the relationship between relative SWA and prosocial preferences was not only present in the first sleep cycle, when the need for sleep and absolute SWA levels were highest, but was similar in all sleep cycles. If the relationship was mainly driven by the sleep need of the brain region, we would have expected a critical role of SWA mainly during the first sleep cycle, which is influenced the most by the sleep pressure that accumulated during the previous wakefulness and thus by state effects (Borbély 1982; Dijk et al., 1987). Finally, prosocial preferences were measured on the day before the sleep EEG measurements took place. This ensured that—should the EEG recording lead to a deteriorated sleep efficiency—this would not influence the behavior in the PGG. As it turned out, the objective sleep efficiency (as measured by actigraphy) on the nights before the EEG measurement was not significantly different from the sleep efficiency on the EEG night (92.1% vs 93.2%). While we have no absolute proof that relative SWA represents a trait-like characteristic, the abovementioned efforts aimed at minimizing state effects. Ultimately, further studies measuring prosociality and sleep physiology longitudinally will be necessary to support our conclusions.

SWA is seen as a physiological marker of sleep depth. We found a correlation between relative SWA values in the TPJ and individual differences in prosocial preferences, suggesting that the local sleep depth specifically in the TPJ may have a crucial impact on prosocial behavior, irrespective of the absolute level of sleep pressure. CSD maps and sLORETA images gave further support for the regional specificity of the association between relative SWA and prosocial preferences in the TPJ. Because SWA is believed to reflect a restorative function (Tononi and Cirelli, 2006; Borbély et al., 2016), we speculate that higher SWA in the right TPJ is indicative of an individual's propensity for prosocial behavior because of local restorative processes. More SWA in the right TPJ might lead to a better restoration of TPJ functions, resulting in a higher capacity for mentalizing and/or perspective-taking, which in turn might lead people to be more prosocially inclined.

Social decision-making is known to be influenced by chronotype or by (sub)optimal time of day (Gunia et al., 2014; Francis et al., 2021). Evening chronotypes, for example, have been shown to be less likely to act prosocially, regardless of whether they have been tested during their matched time (in the evening) or in the morning (Francis et al., 2021). To avoid a confounding factor of chronotype and circadian (mis)timing of our decision-making task, we excluded extreme chronotypes from the present study.

To conclude, we demonstrate that not only sleep duration (as shown by Holbein et al., 2019; Ben Simon et al., 2020; Clark & Dickinson, 2020; Ben Simon et al., 2022) but also sleep depth has an impact on prosocial decisions. Importantly, it depends on where in the brain this happens. Our study offers a first step toward a neural explanation for how sleep patterns explain prosociality by highlighting the crucial role of sleep depth in the right TPJ in prosocial decisions. Our approach therefore improves our understanding of neurobiological mechanisms underlying prosocial preferences and may have implications for future approaches to improve poor perspective-taking and low prosociality. Recent evidence shows that brain stimulation techniques, such as transcranial magnetic stimulation, transcranial direct current stimulation, and auditory closed-loop stimulation, enable the modulation of SWA (Ngo et al., 2013; Bellesi et al., 2014; Sousouri et al., 2021; Lustenberger et al., 2022). Thus, these techniques might be promising tools for boosting SWA in specific areas to potentially remedy dysfunctions and impairments of perspective-taking capacities and other-regarding behavior through targeted interventions.

## Data and Code Availability

Relevant data and code are available at https://github.com/lorenarrgianotti/ProsocialityAndSWA upon publication.
